# Design, Characterization, and Optimization of Controlled Drug Delivery System Containing Antibiotic Drug/s

**DOI:** 10.1155/2016/9024173

**Published:** 2016-08-16

**Authors:** Apurv Patel, Hitesh Dodiya, Pragna Shelate, Divyesh Shastri, Divyang Dave

**Affiliations:** Department of Pharmaceutics and Pharmaceutical Technology, K.B. Institute of Pharmaceutical Education and Research Center, Gandhinagar 382023, India

## Abstract

The objective of this work was design, characterization, and optimization of controlled drug delivery system containing antibiotic drug/s. Osmotic drug delivery system was chosen as controlled drug delivery system. The porous osmotic pump tablets were designed using Plackett-Burman and Box-Behnken factorial design to find out the best formulation. For screening of three categories of polymers, six independent variables were chosen for Plackett-Burman design. Osmotic agent sodium chloride and microcrystalline cellulose, pore forming agent sodium lauryl sulphate and sucrose, and coating agent ethyl cellulose and cellulose acetate were chosen as independent variables. Optimization of osmotic tablets was done by Box-Behnken design by selecting three independent variables. Osmotic agent sodium chloride, pore forming agent sodium lauryl sulphate, and coating agent cellulose acetate were chosen as independent variables. The result of Plackett-Burman and Box-Behnken design and ANOVA studies revealed that osmotic agent and pore former had significant effect on the drug release up to 12 hr. The observed independent variables were found to be very close to predicted values of most satisfactory formulation which demonstrates the feasibility of the optimization procedure in successful development of porous osmotic pump tablets containing antibiotic drug/s by using sodium chloride, sodium lauryl sulphate, and cellulose acetate as key excipients.

## 1. Introduction

Oral controlled drug delivery system can provide continuous delivery of drugs at controlled rate and predictable kinetics throughout the GI transit. Oral controlled drug delivery system targets drug delivery to a specific region for either local or systemic effect throughout the GI transit. This system also gives zero-order release profile [1].

Oral controlled release system can provide better effectiveness in treatment of chronic disease, reduce side effects, and improve patient compliance due to less frequent dosing interval.

Drug release from oral controlled release dosage forms are affected by pH of GI fluid, GI motility, and presence of food in GI tract. Drug release from osmotic drug delivery system is independent of pH and other physiochemical parameters and it is possible to modulate the release characteristic by optimizing the properties of drug and system [2, 3].

Osmotic pressure is used as driving force for osmotic drug delivery systems to release the drug in controlled manner. Osmotic pressure created due to imbibition of fluid from external environment into the dosage form regulates the delivery of drug from osmotic device. Osmotic drug delivery technique is the most interesting and widely acceptable among all other technologies used for the same purpose. Intensive research has been carried out on osmotic systems and several patents are also published. These systems can be used for both routes of administration, that is, oral and parenteral. Oral osmotic systems are known as gastrointestinal therapeutic systems (GITS). Parenteral osmotic drug delivery includes implantable pumps [3].

Dicloxacillin sodium and amoxicillin trihydrate are *β*-Lactam antibiotics. Dicloxacillin sodium and amoxicillin trihydrate have short half-life and high protein binding. The drug that shows linear pharmacokinetics is suitable for oral controlled release tablets and it would be advantageous to slow down its release in GI tract not only to prolong its therapeutic action but also to minimize side effects of drugs.

## 2. Material and Methods 

### 2.1. Materials

Dicloxacillin sodium was obtained as gift sample from Suvik Hitek Pvt. Ltd. (Gandhinagar, India). Amoxicillin trihydrate was obtained as gift sample from Astral Life Care (Mumbai, India). Sodium chloride was purchased from Merck Pharmaceutical (Mumbai, India). Sodium lauryl sulphate was purchased from Bombay Tablet (Gandhinagar, India). Cellulose acetate and PVP K30 were purchased from Chemdyes Corporation (Gujarat). Magnesium stearate and talc were purchased from Suvik Hitek Pvt. Ltd. (Gandhinagar, India).

### 2.2. Differential Scanning Calorimetry (DSC)

DSC studies were carried out for the pure drug, physical mixtures of drug and excipients, and placebo of the porous osmotic pump tablets to study the compatibility. The analysis was performed under nitrogen (nitrogen flow rate 50 mL/min) in order to eliminate oxidative and pyrolytic effects at a standard heating rate of 10°C/min over a temperature range of 50°C–400°C using Universal V4 5A TA instruments.

### 2.3. Preparation of Porous Osmotic Pump Tablet 

#### 2.3.1. Preparation of Core Tablets

Core tablets of dicloxacillin sodium were prepared by wet granulation method. All the ingredients were sieved through # 40 sieve. Individual ingredients, sufficient for a batch of 25 tablets, were weighed on a digital weighing balance as per Table 1. All the ingredients (except PVP K30, magnesium stearate, and talc) were mixed in mortar and pestle using geometric dilution method. The dry blend was granulated with sufficient quantity of PVP K30 which was dissolved in isopropyl alcohol. The powder mass was dried at 60°C in hot air oven for 6 h and passed through # 20 sieve. Then dried granules were mixed with magnesium stearate and talc for 3 min. Tablets were prepared by 9 mm concave die punch set using rotary tablet punching machine [4, 5].

#### 2.3.2. Method of Preparation of Tablet Coat Solution

Cellulose acetate and PEG 400 were added to 3/4th of the total volume of acetone and stirred at 35 rpm using propeller stirrer for half an hour till the solution was clear. Magnesium stearate and coloring agent were triturated thoroughly in a mortar and added to the above solution and stirring continued further. Finally, the volume was made up with acetone [4, 5] (see Table 2).

### 2.4. Coating of the Core Tablets

Tablet coating was done using coating pan apparatus. Speed of coating pan was set at 30 rpm, and inlet air temperature and flow rate were 50°C and 3.2 kg/min, respectively. Spraying rate for coating solution was kept at 4-5 mL/min. Number of tablets per batch was fixed at 50 tablets. Ten tablets of test batch were mixed with 40 dummy tablets. Empty coating pan was run at above set parameters for 5 min. Tablets were loaded to the pan and allowed to gain equilibrium. Coating solution was sprayed at 5 mL/min rate for 2-3 seconds. Coating solution on the tablets was allowed to dry for 5 min and again sprayed. Approximately 100 mL coating solution was used for a batch of 50 tablets.

### 2.5. Characterization of Osmotic Tablet 

#### 2.5.1. Hardness

The fracture strength, which is defined as the force, was required to break a tablet by radial compression and was measured with a Monsanto tablet hardness tester in present study. The mean hardness is calculated and expressed as kg/cm^2^.

#### 2.5.2. Friability

The friability of the tablets was measured in a Roche friabilator. Tablets of a known weight (*w*_0_) or a sample of 10 tablets are dedusted in a drum for a fixed time (100 revolutions) and weighed (*w*) again. Percentage friability was calculated from the loss in weight as given in equation as below. The weight loss should not be more than 1%. Consider

(1) $$\begin{array}{c} \%\;\;Friability=\frac{w_{0}-w}{w_{0}}\times 100. \end{array}$$

#### 2.5.3. Weight Variation Test

To study weight variation, 20 tablets of each formulation were weighed using an electronic balance individually, calculating the average weight, and comparing the individual tablet weights to the average. The tablets meet the IP test if no more than 2 tablets are outside the percentage limit and if no tablet differs by more than 2 times the percentage limit.

#### 2.5.4. Thickness

The thickness of the tablets was determined using a Vernier caliper. 20 tablets were used and mean was calculated. Tablet thickness should not deviate by ±5%.

### 2.6. Determination of Drug Content

Ten tablets were accurately weighed and powdered. A quantity of the powder equivalent to 100 mg of dicloxacillin sodium was weighed accurately and extracted in 100 mL water by shaking for 20 min. After filtration through Whatman filter paper number 1 and sufficient dilution with water, samples were analyzed spectrophotometrically at 273 nm. Amount of drug present was determined from the calibration curve of dicloxacillin sodium [5].

### 2.7. *In Vitro* Drug Release Study

The release rate of dicloxacillin sodium from developed tablets was determined using USP dissolution testing apparatus I (Basket type). The dissolution test was performed using 900 mL 0.1 M HCl (pH 1.2) for 2 hr and then in pH 6.8 phosphate buffer for 10 hr, at 37 ± 0.5°C and 100 rpm. A sample (1 mL) of the solution was withdrawn from the dissolution apparatus hourly for 12 h, and the samples were replaced with fresh dissolution medium. The samples were passed through Whatman filter paper after dilution and the absorption of these solutions was measured at 273 nm. The cumulative percentage drug release was calculated.

### 2.8. Curve Fitting Analysis

For the determination of the drug release kinetics from the porous osmotic pump tablet, the in vitro release data were analyzed by zero-order, first-order, Higuchi, and Korsmeyer and Peppas equations [6].

### 2.9. Zero-Order Release Kinetics

To study the zero-order release kinetics the release data was fitted into the following equation:

(2) $$\begin{array}{c} \frac{dQ}{dt}=K_{0}, \end{array}$$

where “*Q*” is the amount of drug release, “*K*_0_” is the zero-order release rate constant, and “*t*” is the release time. The graph is plotted percentage cumulative drug release (% CDR) versus time.

### 2.10. First-Order Release Kinetics

To study the first-order release kinetics the release rate data are fitted into the following equation:

(3) $$\begin{array}{c} \frac{dQ}{dt}=K_{1}Q, \end{array}$$

where “*Q*” is the fraction of drug release, “*K*_1_” is the first-order release rate constant, and “*t*” is the release time.

### 2.11. Higuchi Release Model

To study the Higuchi release model the release rate data are fitted into the following equation:

(4) $$\begin{array}{c} Q=K_{H}t^{1/2}, \end{array}$$

where “*Q*” is the fraction of drug release, “*K*_*H*_” is the release rate constant, and “*t*” is the release time. The graph is plotted as % CDR versus square root of time.

### 2.12. Korsmeyer and Peppas Kinetics

To study the Korsmeyer and Peppas release kinetics the release rate data are fitted into the following equation:

(5) $$\begin{array}{c} \frac{M_{t}}{M_{\infty }}=K_{KP}t^{n}, \end{array}$$

where *M*_*t*_/*M*_*∞*_ is the fraction of drug release, “*K*_KP_” is the release rate constant, “*t*” is the release time, and “*n*” is the diffusion exponent related to mechanism of drug release. The graph is plotted as log % CDR versus log time [6].

### 2.13. Selection of Polymers by Plackett-Burman Factorial Design

A Plackett-Burman design was adopted to selection of polymers of different category. In this design six factors were evaluated. Hence by applying Plackett-Burman factorial design, influence of six independent variables, osmotic agents (NaCl, MCC), pore forming agents (SLS, sucrose), and coating agents (cellulose acetate, ethyl cellulose), was studied over three dependent variables' drug release at 2 hr, 6 hr, and 12 hr and was checked [6, 7]. Formulation of osmotic tablets of factorial batches is shown in Tables 3 and 4.

### 2.14. Optimization of Osmotic Tablet by Box-Behnken Factorial Design

In this optimization technique, the desirability approach was used to generate the optimum settings for the formulation. From the trial batches, three independent variables were found to affect drug release significantly. Concentration of coating agent (NaCl) and pore forming agent (SLS) and concentration of coating agent (cellulose acetate) were taken as independent variables [8, 9]. For the optimized formulation, the drug release at 2 hr, 6 hr, and 12 hr and release exponent (*n*) were kept in target. Formulation of osmotic tablets of factorial batches is shown in Tables 5 and 6.

### 2.15. Effect of pH on Drug Release

The optimized formulation of porous osmotic pump tablets was tested for the effect of pH on drug release. The best formulations were undergone in dissolution studies in 0.1 N HCl, 6.8 pH phosphate buffer, 7.5 pH phosphate buffer, and distilled water in rotation speed of 100 rpm and 37 ± 0.5°C using USP dissolution test apparatus type 1.

### 2.16. Effect of Agitation Intensity Drug Release

The optimized formulation of matrix and porous osmotic pump tablets are tested for the effect of agitation intensity on drug release. The best formulations are undergone in dissolution studies by maintaining different rotation speed of 50, 100, and 150 rpm and at 37 ± 0.5°C in 7.5 pH phosphate buffer for 8 h using USP dissolution test apparatus type 1.

### 2.17. Stability Studies

The stability studies were carried out as per the ICH and WHO guidelines of stability testing. Optimized formulations were kept inside the stability chamber maintained at 45°C and 75% RH for the period of 30 days. At the end of the stability study period, samples were analyzed for parameters like physical characteristics, drug content, and in vitro drug release.

## 3. Result and Discussion

### 3.1. Drug Polymer Compatibility Studies Using DSC

**Figure 1:**
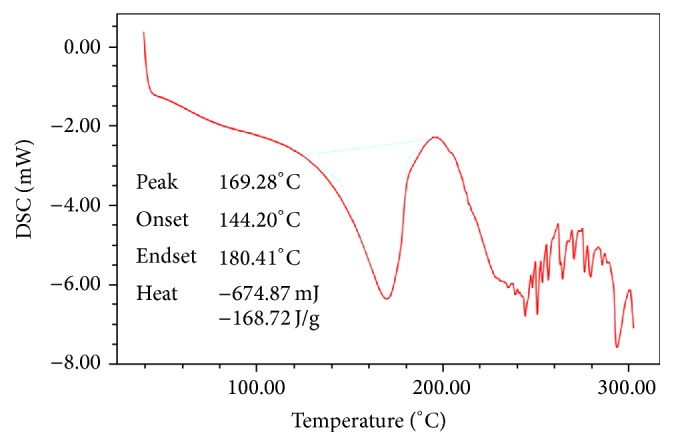
DSC spectra of pure drug (dicloxacillin sodium).

**Figure 2:**
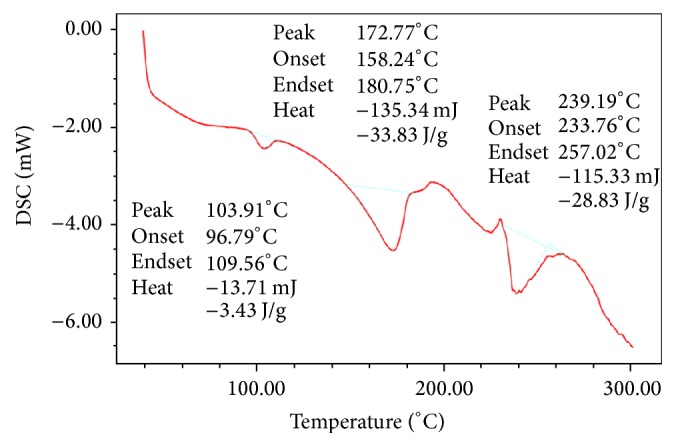
DSC spectra of drug and polymers.

DSC thermograms of pure drug (dicloxacillin sodium) and physical mixtures of drug and excipients (NaCl, SLS, and CA) were studied for their interactions. It was observed that there was no significant drug polymer interaction observed among drug, NaCl, SLS, and CA even at higher temperature. From DSC study, we can see that there is no change in drug's melting peak (169.28°C–172.77°C) after the preparation of mixture. There is the no interaction between drug and excipient shown in this study. So, we can conclude that drug is compatible with all polymers. DSC thermograms were shown in Figures 1 and 2.

### 3.2. Screening of Polymers by Plackett-Burman Factorial Design

#### 3.2.1. Physicochemical Properties

Twelve batches were prepared for screening of polymers. The mean values of hardness, friability, thickness, weight, and drug content of prepared porous osmotic pump tablets are shown in Table 7.

#### 3.2.2. *In Vitro *Dissolution Study

**Figure 3:**
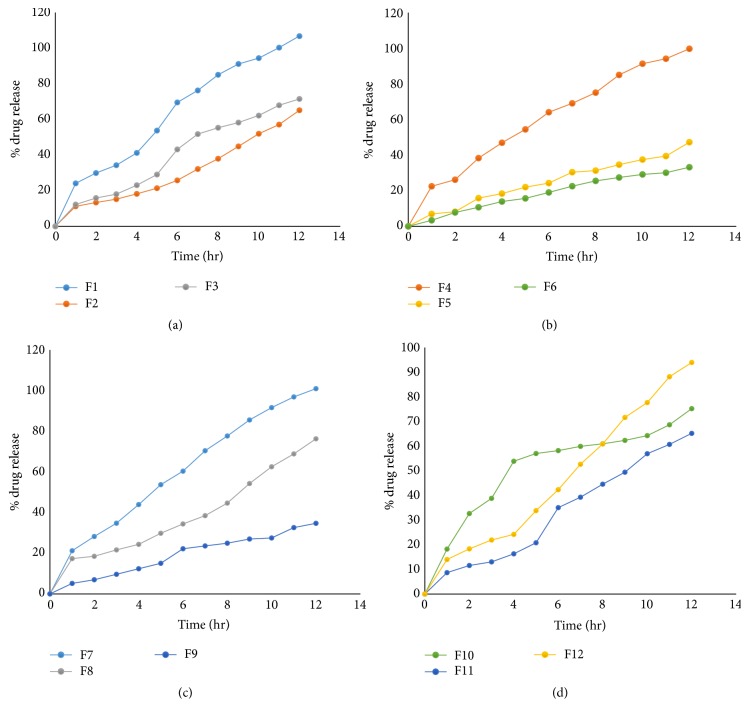
(a) Drug release profile for batches F1 to F3. (b) Drug release profile for batches F4 to F6. (c) Drug release profile for batches F7 to F9. (d) Drug release profile for batches F10 to F12.

To study all the possible combinations of all factors at all levels, a six-factor, two-level Plackett-Burman factorial design was constructed and conducted in a fully randomized order. Six factors, NaCl(*X*1), MCC(*X*2), SLS(*X*3), sucrose(*X*4), EC(*X*5), and CA(*X*6), were selected as independent variables. Twelve batches were prepared to study Plackett-Burman factorial design for osmotic tablets. Two checkpoint batches were also evaluated to validate the design. The dependent variables (responses) studied were % drug release after 1 hr, 6 hr, and 12 hr of dissolution. Results of the drug release profile obtained for osmotic tablets are shown in Figures 3(a), 3(b), 3(c), and 3(d).

*Effect of formulation variable on drug release* at 1 hr, 6 hr, and 12 hr was carried out using Design-Expert Software (Version 7.1.6, Stat-Ease Inc., Minneapolis, MN).

**Figure 4:**
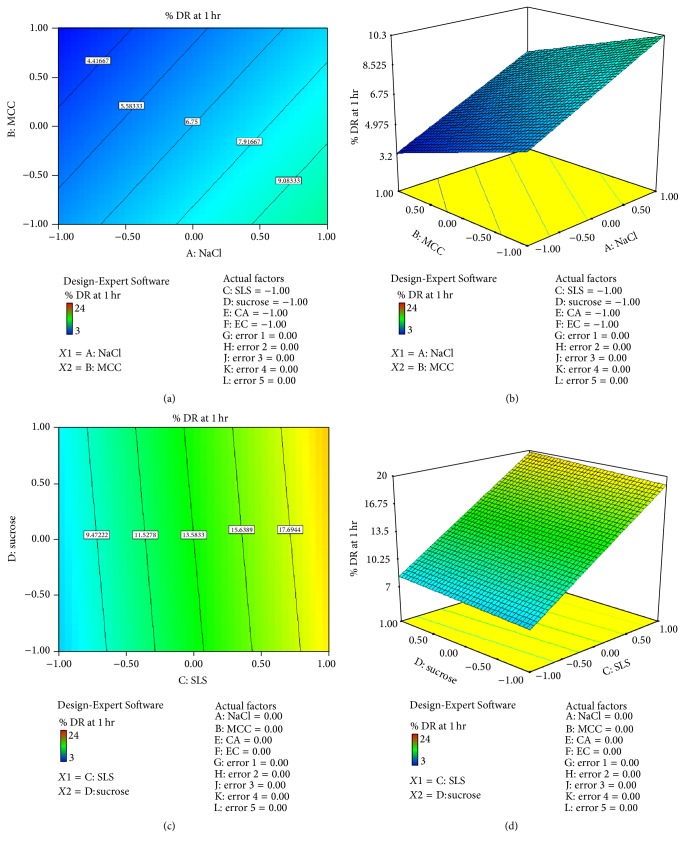
(a) Contour plot for response *Y*1 (drug release at 1 hr) (for *X*1  and  *X*2). (b) 3D surface plot of response *Y*1 (drug release at 1 hr) (for *X*1  and  *X*2). (c) Contour plot for response *Y*1 (drug release at 1 hr) (for *X*3  and  *X*4). (d) 3D surface plot of response *Y*1 (drug release at 1 hr) (for *X*3  and  *X*4).

*Effect of Formulation Variable on Drug Release at 1 hr (Y1)*. From the equation, factor value of *X*1 was +2.08 and *X*2 was −1.41 indicating that *X*1 had more effect on drug release than *X*2. Factor value of *X*3 was +5.75 and *X*4 was +0.41 indicating that *X*3 had more effect on drug release than *X*4. Factor value of *X*5 was +0.08 and *X*6 was +0.53 indicating that *X*6 had more effect on drug release than *X*5. Positive sign of *X*1, *X*3 , and   *X*6 indicates positive effect on drug release. Figures 4(a)–4(d) show contour plot and 3D surface plot for *Y*1 suggesting effect of variables as described above. Consider

(6) Y1=2.08X1−1.41X2+5.75X3+0.41X4+0.08X5+0.53X6.

The relationship between formulation variables (*X*1 and *X*2) and *Y*1 was further elucidated using 3D surface plot. From Figure 4(b) it can be concluded that factor NaCl(*X*1) had more osmotic effect on drug release while MCC(*X*2) had no significant effect on drug release.

The relationship between formulation variables (*X*3 and *X*4) and *Y*1 was further elucidated using 3D surface plot. From Figure 4(d) it can be concluded that factor SLS(*X*3) had more pore forming effect on drug release while sucrose(*X*4) had no significant effect on drug release.

**Figure 5:**
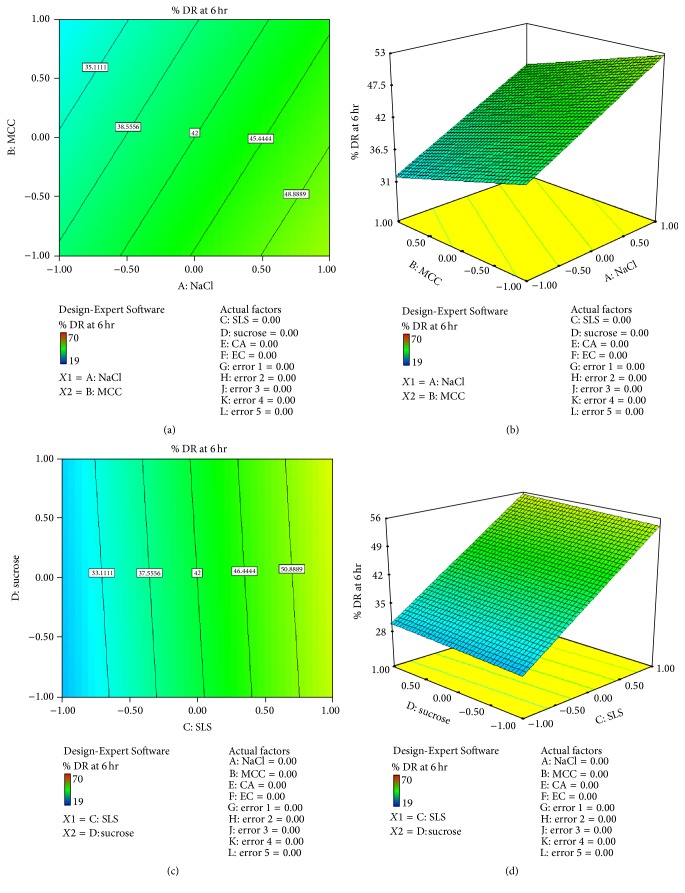
(a) Contour plot for response *Y*2 (drug release at 6 hr) (for *X*1  and  *X*2). (b) 3D surface plot of response *Y*2 (drug release at 6 hr) (for *X*1  and  *X*2). (c) Contour plot for response *Y*2 (drug release at 6 hr) (for *X*3 and  *X*4). (d) 3D surface plot of response *Y*2 (drug release at 6 hr) (for *X*3  and  *X*4).

*Effect of Formulation Variable on Drug Release at 6 hr (Y*2*). *From the equation, factor value of *X*1 was +6.66 and *X*2 was −3.66 indicating that *X*1 had more effect on drug release than *X*2. Factor value of *X*3 was +12.66 and *X*4 was +0.66 indicating that *X*3 had more effect on drug release than *X*4. Factor value of *X*5 was −0.16 and *X*6 was +3.83 indicating that *X*6 had more effect on drug release than *X*5. Positive sign of *X*1, *X*3, and  *X*6 indicates positive effect on drug release. Figures 5(a)–5(d) show contour plot and 3D surface plot for *Y*2 suggesting effect of variables as described above. Consider

(7) Y2=6.66X1−3.66X2+12.66X3+0.66X4−0.16X5+3.83X6.

The relationship between formulation variables (*X*1 and *X*2) and *Y*2 was further elucidated using 3D surface plot. From Figure 5(b) it can be concluded that factor NaCl(*X*1) had more osmotic effect on drug release while MCC(*X*2) had no significant effect on drug release.

The relationship between formulation variables (*X*3 and *X*4) and *Y*2 was further elucidated using 3D surface plot. From Figure 5(d) it can be concluded that factor SLS(*X*3) had more pore forming effect on drug release while sucrose(*X*4) had no significant effect on drug release.

**Figure 6:**
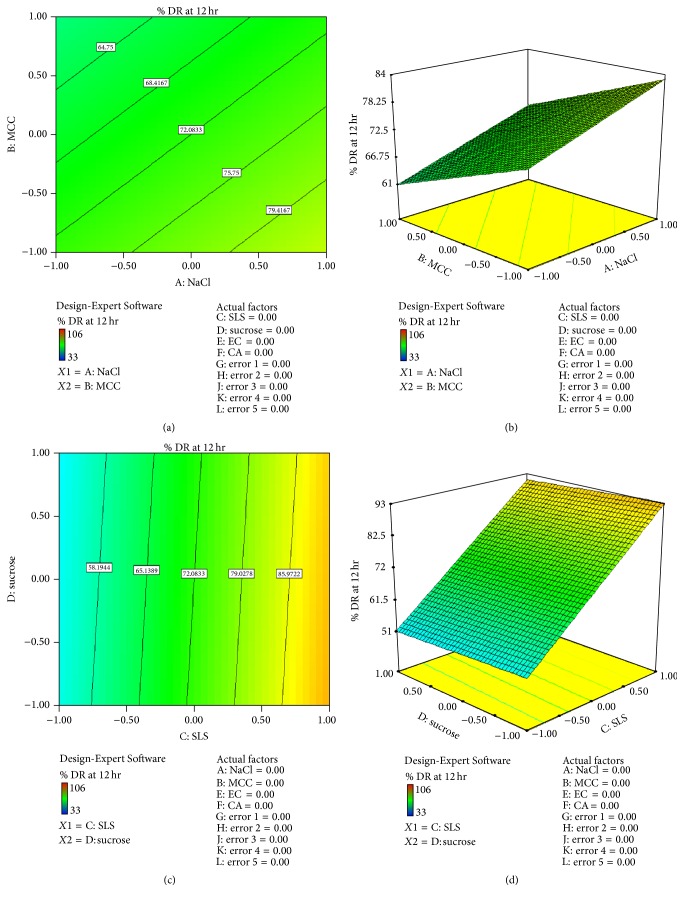
(a) Contour plot for response *Y*3 (drug release at 12 hr) (for *X*1  and  *X*2). (b) 3D surface plot of response *Y*3 (drug release at 12 hr) (for *X*1  and  *X*2). (c) Contour plot for response *Y*3 (drug release at 12 hr) (for *X*3  and  *X*4). (d) 3D surface plot of response *Y*3 (drug release at 12 hr) (for *X*3  and  *X*4).

*Effect of Formulation Variable on Drug Release at 12 hr (Y*3). From the equation, factor value of *X*1 was +5.08 and *X*2 was −5.19 indicating that *X*1 had more effect on drug release than *X*2. Factor value of *X*3 was +19.75 and *X*4 was −1.08 indicating that *X*3 had more effect on drug release than *X*4. Factor value of *X*5 was +2.41 and *X*6 was +7.25 indicating that *X*6 had more effect on drug release than *X*5. Positive sign of *X*1, *X*3, and  *X*6 indicates positive effect on drug release. Figures 6(a)–6(d) show contour plot and 3D surface plot for *Y*3 suggesting effect of variables as described above. Consider

(8) Y3=5.08X1−5.19X2+19.75X3−1.083X4+2.41X5+7.25X6.

The relationship between formulation variables (*X*1 and *X*2) and *Y*3 was further elucidated using 3D surface plot. From Figure 6(b) it can be concluded that factor NaCl(*X*1) had more osmotic effect on drug release while MCC(*X*2) had no significant effect on drug release.

The relationship between formulation variables (*X*3 and *X*4) and *Y*3 was further elucidated using 3D surface plot. From Figure 6(d) it can be concluded that factor SLS(*X*3) had more pore forming effect on drug release while sucrose(*X*4) had no significant effect on drug release.

### 3.3. Optimization of Osmotic Tablet by Box-Behnken Factorial Design

#### 3.3.1. Physiochemical Parameter

15 batches were prepared for optimization of osmotic tablets. Tablets were evaluated for uniformity of weight, uniformity of contents, tablet thickness and diameter, and hardness and friability. Results of the physiochemical tests obtained are shown in Table 8.

#### 3.3.2. *In Vitro *Dissolution Study

**Figure 7:**
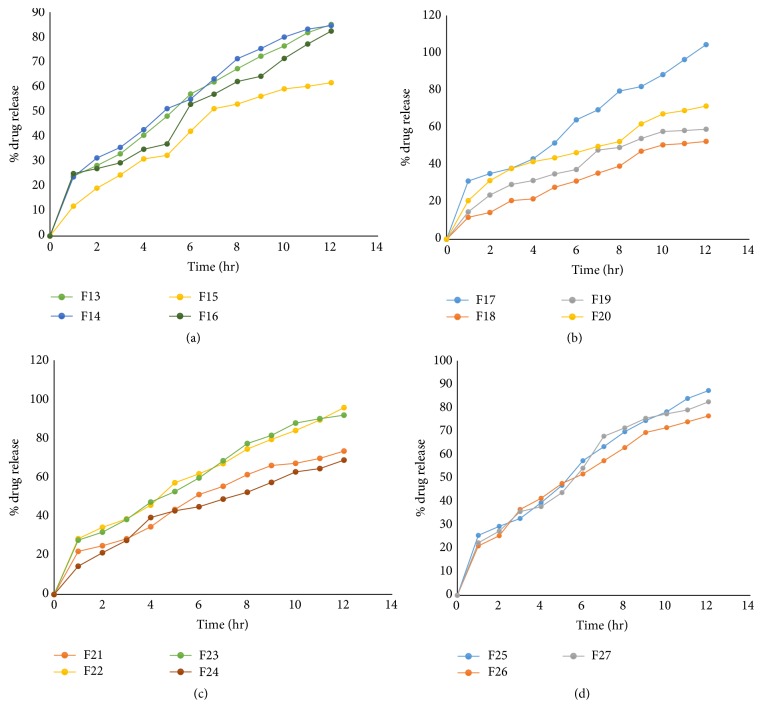
(a) Drug release profile for batches (F13–F16). (b) Drug release profile for batches (F17–F20). (c) Drug release profile for batches (F21–F24). (d) Drug release profile for batches (F25–F27).

To study all the possible combinations of all factors at all levels, a three-factor, three-level Box-Behnken factorial design was constructed and conducted in a fully randomized order. Three factors, NaCl(*X*1), SLS(*X*2), and CA(*X*3), were selected as independent variables. 15 batches were prepared to study Box-Behnken factorial design for osmotic tablets. Two checkpoint batches were also evaluated to validate the design. The dependent variables (responses) studied were % drug release after 1 hr, 6 hr, and 12 hr of dissolution. Results of the drug release profile obtained for osmotic tablets are shown in Figures 7(a), 7(b), 7(c), and 7(d).

Figure 7(a) contains dissolution profile for batches F13–F16.  Figure 7(b) contains dissolution profile for batches F17–F20.  Figure 7(c) contains dissolution profile for batches F21–F124.  Figure 7(d) contains dissolution profile for batches F25–F127.

**Figure 8:**
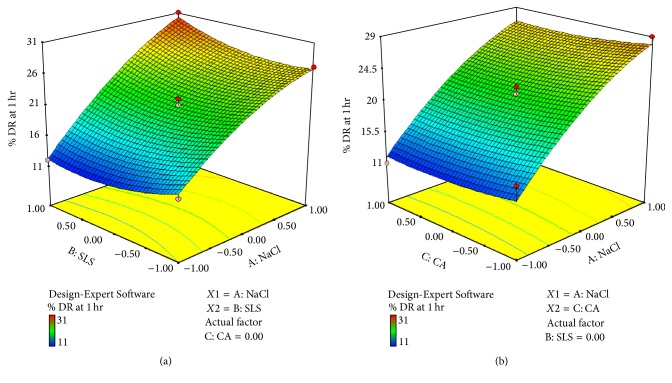
(a) 3D surface plot of response *Y*1 (drug release at 1 hr) (for *X*1  and  *X*2). (b) 3D surface plot of response *Y*1 (drug release at 1 hr) (for *X*1  and  *X*3).

*Effect of Formulation Variable on Drug Release at 1 hr (Y1)*. Equation shows that coefficients *b*_1_ and *b*_2_ bear a positive sign and *b*_3_ bears a negative sign and coefficient value for *X*1 is 7.50, *X*2 is 0.25, and *X*3 is −0.50. So it indicates that *X*1 has more effect on drug release than *X*2 and *X*3. Figures 8(a) and 8(b) show cube and 3D surface plot for *Y*1 suggesting effect of variables as described above. Consider

(9) Y1=7.50X1+0.25X2−0.50X3+1.50X1X2+0.00X1X3+0.50X2X3−1.92X12+1.58X22+0.58X32.

The relationship between formulation variables (*X*1, *X*2, and *X*3) and *Y*1 was further elucidated using cube and 3D surface plot. From Figure 8(a) it can be concluded that factor Nacl(*X*1) has more effect on drug release than SLS(*X*2). From Figure 8(b) it can be concluded that factor *X*3 (Coating agent) has negative effect on drug release. As we increase the level of *X*3, it decreases drug release.

**Figure 9:**
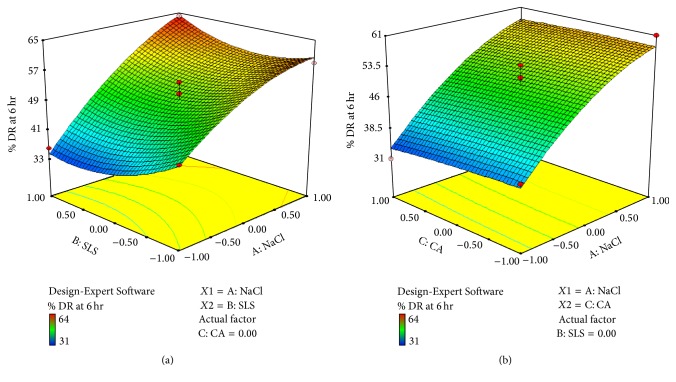
(a) 3D surface plot of response *Y*2 (drug release at 6 hr) (for *X*1  and  *X*2). (b) 3D surface plot of response *Y*2 (drug release at 6 hr) (for *X*1  and  *X*3).

*Effect of Formulation Variable on Drug Release at 6 hr (Y2). *Equation shows that coefficients *b*_1_ and *b*_2_ bear a positive sign and *b*_3_ bears a negative sign and coefficient value for *X*1 is 11.62, *X*2 is 0.38, and *X*3 is −0.75. So it indicates that *X*1 has more effect on drug release than *X*2 and *X*3. Figures 9(a) and 9(b) show cube and 3D surface plot for *Y*2 suggesting effect of variables as described above. Consider

(10) Y1=11.62X1+0.38X2−0.75X3+3.25X1X2+0.50X1X3+0.00X2X3−3.54X12+3.96X22−0.96X32.

The relationship between formulation variables (*X*1, *X*2, and *X*3) and *Y*2 was further elucidated using cube and 3D surface plot. From Figure 9(a) it can be concluded that factor NaCl(*X*1) has more effect on drug release than SLS(*X*2). From Figure 9(b) it can be concluded that factor *X*3 (Coating agent) has negative effect on drug release. As we increase the level of *X*3, it decreases drug release.

**Figure 10:**
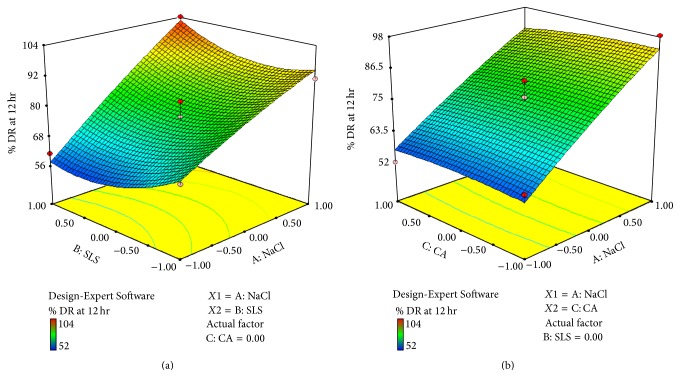
(a) 3D surface plot of response *Y*3 (drug release at 12 hr) (for *X*1  and  *X*2). (b) 3D surface plot of response *Y*3 (drug release at 12 hr) (for *X*1  and  *X*3).

*Effect of Formulation Variable on Drug Release at 12 hr (Y3). *Equation shows that coefficients *b*_1_ and *b*_2_ bear a positive sign and *b*_3_ bears a negative sign and coefficient value for *X*1 is 17.50, *X*2 is 0.88, and *X*3 is −0.80. So it indicates that *X*1 has more effect on drug release than *X*2 and *X*3. Figures 10(a) and 10(b) show cube and 3D surface plot for *Y*2 suggesting effect of variables as described above. Consider

(11) Y3=17.50X1+0.88X2−0.80X3+5.00X1X2−1.00X1X3+2.25X2X3−1.29X12+5.96X22−1.04X32.

The relationship between formulation variables (*X*1, *X*2, and *X*3) and *Y*3 was further elucidated using cube and 3D surface plot. From Figure 10(a) it can be concluded that factor NaCl(*X*1) has more effect on drug release than SLS(*X*2). From Figure 10(b) it can be concluded that factor *X*3 (coating agent) has negative effect on drug release. As we increase the level of *X*3, it decreases drug release.

### 3.4. Selection of Optimized Batch

Selection of best batch was carried out using* Design-Expert Software (Version 7.1.6, Stat-Ease Inc., Minneapolis, MN)*. After statistical analysis the desirability function was applied to select the best batch. The desirable values selected for dependent variables *Y*1, *Y*2, and  *Y*3 are given in Table 9.

Desirable value range selected that was 5% varies from optimum value.

**Figure 11:**
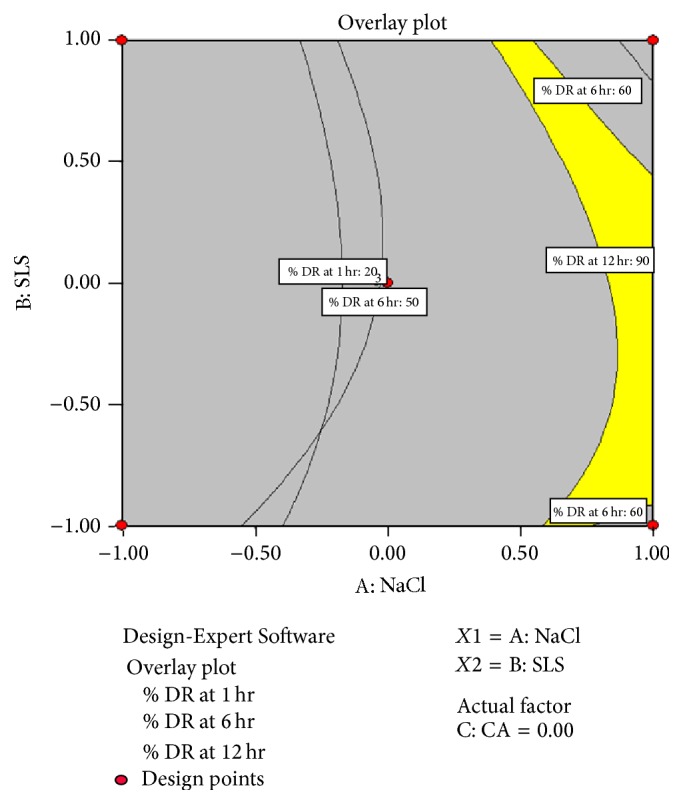
Overlay plot of *Y*1, *Y*2, and  *Y*3.

*Batch F22* came closest to satisfying all the selection criteria. The results were further reinstated using the overlay plot in Figure 11. The yellow region of the plot indicates the area where all the selection criteria are satisfied.* Batch F22* falls in this yellow area, indicating the formulation having amount of osmotic agent (150 mg), pore forming agent (15 mg), and coating agent (2%) that possessed the desirable characteristics.

### 3.5. So F22 Batch Was Selected as Optimized Batch

#### 3.5.1. Effect of pH on Drug Release

When formulation F22 was subjected to in vitro release studies in buffers with different pH and distilled water, no significant differences in the release profiles were seen compared to that in phosphate buffer pH 6.8. Thus the fluid in different parts of the GI tract will scarcely affect drug release from the osmotic system.

#### 3.5.2. Effect of Agitation Intensity on Drug Release

The release profile of dicloxacillin sodium from the optimized formulation F22 was independent of the agitational intensity of the release media.

## 4. Osmotic Tablet of Amoxicillin Trihydrate

**Figure 12:**
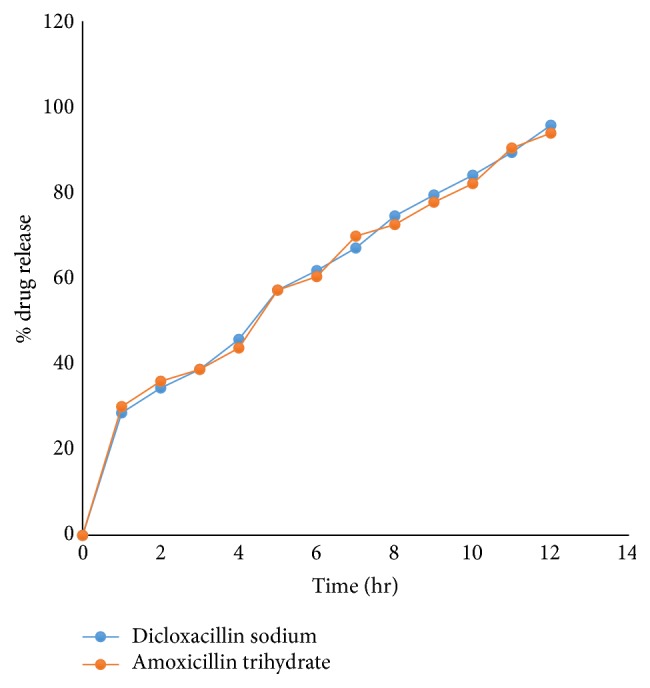
Drug release data of dicloxacillin sodium and amoxicillin trihydrate.

Optimized batch of amoxicillin trihydrate was prepared using F22 batch composition of Box-Behnken design batches. From the drug release data and also release pattern shown in Figure 12 it can be concluded that there is no significant difference between two drug release profiles.

## 5. Release Kinetics and Release Mechanism

Six kinetic models were used for controlled release curve fitting to select the most appropriate model. The dissolution data for optimized batch was fitted to the zero-order, first-order, Higuchi, Hixson-Crowell, Korsmeyer-Peppas, and Weibull models. Best fitting model was selected on the basis of highest correlation coefficient and lowest *F* value. Comparative statistical parameters for all the models were obtained as shown in Table 11. Drug release mechanism was explored on the basis of release exponent (*n*) value.

Model fitting results revealed that the Korsmeyer-Peppas model was best fitted to the release kinetics (*r* ^2^ = 0.9960, highest; *F* = 6.8629, lowest). Higuchi model was also close to the Korsmeyer-Peppas model. Hence *F* test was performed for both models. It revealed significant difference between the two models. Hence Korsmeyer-Peppas model was finally selected as best fitted model. Release exponent *n* was found to be 0.580, indicating that the drug was released from the formulation by* anomalous (non-Fickian) mechanism*.

## 6. Stability Study

**Figure 13:**
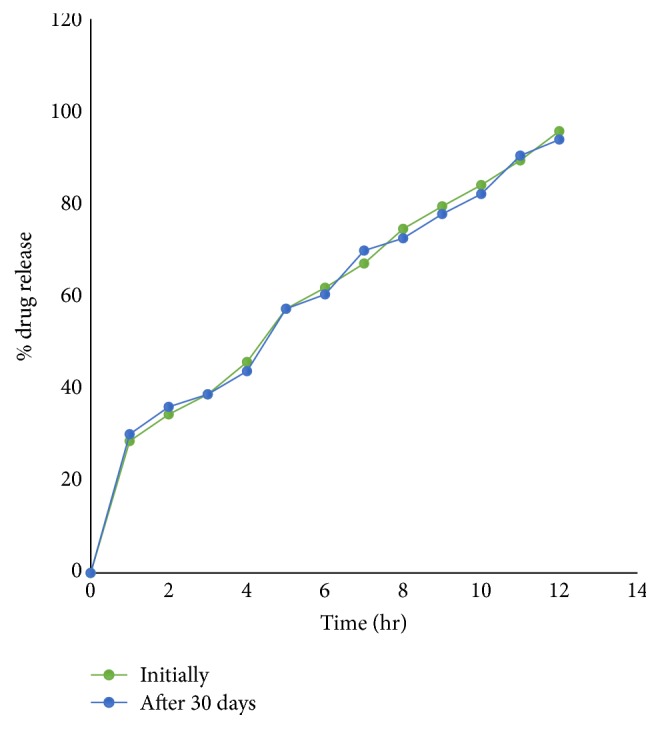
Drug release data for stability study of osmotic tablet (F22).

After the 1-month storage of formulation F22, values of all parameters like hardness, diameter, thickness, % drug content, and friability were checked periodically and found to be almost similar to the initial values. The drug profile was similar to the initial profile shown in Figure 13. There was not any significant change in any value and also no changes in the physical appearance. So it can be said that formulation is stable (see Table 12).

## 7. Conclusion 

The observed independent variables were found to be very close to predicted values of optimized formulation which demonstrates the feasibility of the optimization procedure in successful development of porous osmotic pump tablets containing dicloxacillin sodium and amoxicillin trihydrate as model drug by using sodium chloride (150 mg) as osmotic agent, sodium lauryl sulphate (15 mg) as pore former, cellulose acetate (2%) as coating agent, and control membrane permeability. Batch F22 was selected as optimized batch. Stability studies also revealed that optimized formulation is stable.

From the comparison of dissolution profile of optimized batch for both drugs (dicloxacillin sodium and amoxicillin trihydrate) it can be concluded that there was no significance difference in drug release observed, so it concludes that porous osmotic pump tablets of antibiotic drugs were successfully developed (see Table 10).

## Figures and Tables

**Table 1:** Formulation of osmotic tablet.

Ingredient	Weight (mg)
Dicloxacillin sodium	244
Sodium chloride	100
MCC	60
Sodium lauryl sulfate	15
Sucrose	60
PVP K30	15
Magnesium stearate	3
Talc	3

Total weight of one tablet = 500 mg.

Number of tablets per batch = 20.

**Table 2:** Composition of coating solvent.

Ingredient	Composition
Cellulose acetate	2% w/v
PEG 400	2% v/v
TiO_2_	0.2% w/v
Coloring agent	0.2% w/v
Acetone	Up to 100 mL

**Table 3:** Formulation of osmotic tablet (F1–F6).

Ingredient	Batch number					
	F1	F2	F3	F4	F5	F6
Dicloxacillin sodium (mg)	244	244	244	244	244	244
NaCl (mg)	150	150	150	150	150	50
MCC (mg)	30	30	90	30	90	30
SLS (mg)	20	10	10	20	10	10
Sucrose (mg)	90	30	30	90	90	30
PVP K30 (mg)	15	15	15	15	15	15
EC (%)	4	4	2	2	4	2
CA (%)	2	2	4	4	4	2
PEG 400 (%)	2	2	2	2	2	2
Magnesium stearate (mg)	3	3	3	3	3	3
Talc (mg)	3	3	3	3	3	3
Coloring agent	q.s	q.s	q.s	q.s	q.s	q.s

**Table 4:** Formulation of osmotic tablet (F7–F12).

Ingredient	Batch number					
	F7	F8	F9	F10	F11	F12
Dicloxacillin sodium (mg)	244	244	244	244	244	244
NaCl (mg)	50	50	50	150	50	50
MCC (mg)	30	90	90	90	30	90
SLS (mg)	20	20	10	20	10	20
Sucrose (mg)	50	90	90	30	90	30
PVP K30 (mg)	15	15	15	15	15	15
EC (%)	4	2	4	2	2	4
CA (%)	4	2	2	2	4	2
PEG 400 (%)	2	2	2	2	2	2
Magnesium stearate (mg)	3	3	3	3	3	3
Talc (mg)	3	3	3	3	3	3
Coloring agent	q.s	q.s	q.s	q.s	q.s	q.s

**Table 5:** Formulation of osmotic tablet (F13–F20).

Ingredient	Batch number							
	F13	F14	F15	F16	F17	F18	F19	F20
Dicloxacillin sodium (mg)	244	244	244	244	244	244	244	244
NaCl (mg)	100	100	50	100	150	50	50	100
SLS (mg)	10	10	20	20	20	15	15	20
PVP K30 (mg)	15	15	15	15	15	15	15	15
CA (%)	4	2	3	4	3	4	2	2
PEG 400 (%)	2	2	2	2	2	2	2	2
Magnesium stearate (mg)	3	3	3	3	3	3	3	3
Talc (mg)	3	3	3	3	3	3	3	3
Coloring agent	q.s	q.s	q.s	q.s	q.s	q.s	q.s	q.s

**Table 6:** Formulation of osmotic tablet (F21–F27).

Ingredient	Batch number						
	F21	F22	F23	F24	F25	F26	F27
Dicloxacillin sodium (mg)	244	244	244	244	244	244	244
NaCl (mg)	100	150	150	50	150	100	100
SLS (mg)	20	15	10	10	15	15	15
PVP K30 (mg)	15	15	15	15	15	15	15
CA (%)	2	2	3	3	4	3	3
PEG 400 (%)	2	2	2	2	2	2	2
Magnesium stearate (mg)	3	3	3	3	3	3	3
Talc (mg)	3	3	3	3	3	3	3
Coloring agent	q.s	q.s	q.s	q.s	q.s	q.s	q.s

**Table 7:** Physiochemical parameters of factorial batches.

Batch code	Diameter (mm)	Thickness (mm)	Hardness (kg/cm^2^)	% friability	Uniformity of weight (mg)	% drug content (% w/w)
F1	10.0 ± 0.05	5.2 ± 0.5	6.3 ± 0.2	0.54	540 ± 2.7	103.2 ± 2.1
F2	10.1 ± 0.05	4.9 ± 0.5	6.8 ± 0.4	0.39	470 ± 4.8	99.3 ± 3.6
F3	10.1 ± 0.05	5.1 ± 0.2	6.5 ± 0.5	0.72	530 ± 2.9	98.2 ± 4.3
F4	10.1 ± 0.05	5.2 ± 0.5	6.3 ± 0.7	0.43	540 ± 3.1	97.3 ± 4.7
F5	10.0 ± 0.05	4.6 ± 0.4	6.8 ± 0.3	0.11	385 ± 3.4	100.3 ± 2.2
F6	10.0 ± 0.05	5.4 ± 0.3	6.9 ± 0.2	0.47	590 ± 1.8	99.5 ± 1.4
F7	10.1 ± 0.05	4.7 ± 0.5	6.8 ± 0.2	0.52	380 ± 4.4	101.4 ± 1.2
F8	10.0 ± 0.05	4.9 ± 0.5	6.8 ± 0.5	0.65	500 ± 2.5	96.3 ± 3.3
F9	10.0 ± 0.05	4.8 ± 0.4	6.5 ± 0.2	0.83	430 ± 3.7	98.4 ± 1.4
F10	10.0 ± 0.05	5.2 ± 0.2	6.8 ± 0.4	0.49	540 ± 1.2	99.2 ± 3.8
F11	10.1 ± 0.05	4.8 ± 0.5	6.8 ± 0.5	0.57	430 ± 5.1	97.7 ± 2.6
F12	10.0 ± 0.05	4.8 ± 0.5	6.5 ± 0.3	0.34	440 ± 2.9	97.2 ± 1.3

All values are mean of three readings.

**Table 8:** Physiochemical parameters of factorial batches.

Batch code	Diameter	Thickness	Hardness	% friability	Weight variation	% drug content
F13	10.1 ± 0.05	4.6 ± 0.5	6.8 ± 0.3	0.54	375 ± 2.6	101.2 ± 2.3
F14	10.1 ± 0.05	4.6 ± 0.3	6.4 ± 0.3	0.39	375 ± 4.8	98.3 ± 3.6
F15	10.0 ± 0.05	4.2 ± 0.2	6.5 ± 0.3	0.72	335 ± 2.9	99.2 ± 4.1
F16	10.0 ± 0.05	4.7 ± 0.5	6.3 ± 0.5	0.43	384 ± 3.1	95.3 ± 4.4
F17	10.1 ± 0.05	5.1 ± 0.4	6.8 ± 0.2	0.11	435 ± 3.4	103.3 ± 2.5
F18	10.0 ± 0.05	4.2 ± 0.3	6.9 ± 0.3	0.47	330 ± 1.8	97.5 ± 1.7
F19	10.0 ± 0.05	4.3 ± 0.5	6.8 ± 0.5	0.52	330 ± 4.4	101.4 ± 1.2
F20	10.1 ± 0.05	4.7 ± 0.5	6.8 ± 0.4	0.65	380 ± 2.5	98.3 ± 3.5
F21	10.1 ± 0.05	4.8 ± 0.4	6.5 ± 0.2	0.83	385 ± 3.7	99.4 ± 1.4
F22	10.0 ± 0.05	5.0 ± 0.2	6.8 ± 0.2	0.49	430 ± 1.2	97.2 ± 3.8
F23	10.1 ± 0.05	4.9 ± 0.5	6.9 ± 0.3	0.57	426 ± 5.1	98.7 ± 2.6
F24	10.0 ± 0.05	5.2 ± 0.5	6.5 ± 0.2	0.34	440 ± 2.9	98.2 ± 1.3
F25	10.1 ± 0.05	4.2 ± 0.4	6.6 ± 0.5	0.43	325 ± 1.3	99.4 ± 5.4
F26	10.1 ± 0.05	4.7 ± 0.1	6.9 ± 0.2	0.61	380 ± 3.4	98.5 ± 1.7
F27	10.0 ± 0.05	4.7 ± 0.5	6.8 ± 0.1	0.53	380 ± 2.7	99.9 ± 2.3

All values are mean of three readings.

**Table 9:** Desirable values selected for dependent variables.

Dependent variables	Desirable values	
	Lower limit	Upper limit
*Y*1 (% drug release at 1 hr)	20	30
*Y*2 (% drug release at 6 hr)	50	60
*Y*3 (% drug release at 12 hr)	90	100

**Table 10:** Comparison of drug release profiles of dicloxacillin sodium and amoxicillin trihydrate.

Time (hr)	Dicloxacillin sodium	Amoxicillin trihydrate
0	0	0
1	28.56	30.07
2	34.38	35.98
3	38.72	38.72
4	45.72	43.72
5	57.23	57.23
6	61.81	60.37
7	67.04	69.84
8	74.53	72.53
9	79.45	77.75
10	84.02	82.09
11	89.39	90.39
12	95.72	93.88

**Table 11:** Kinetic modeling of drug release.

Parameter	Kinetic model					
	Zero-order	First-order	Higuchi	Hixson-Crowell	Korsmeyer-Peppas	Weibull
Sum of residuals	1314.416	346.8983	205.599	336.2533	**75.4920**	258.9369
Correlation coefficient (*r*)	0.9781	0.9821	0.9905	0.9877	**0.9960**	0.9860
*R* square (*r* ^2^)	0.8595	0.9629	0.9780	0.9635	**0.9918**	0.9719
*F*	109.534	28.9081	17.1332	28.0211	**6.8629**	25.8936

Model fitting results revealed that the Korsmeyer-Peppas model was best fitted to the release kinetics (*r* ^2^ = 0.9960, highest, *F* = 6.8629, lowest). Higuchi model was also close to the Korsmeyer-Peppas model. Hence *F* test was performed for both models. It revealed significant difference between two models. Hence Korsmeyer-Peppas model was finally selected as best fitted model. Release exponent *n* was found to be 0.580, indicating that the drug was released from the formulation by *anomalous (non-Fickian) mechanism*.

**Table 12:** Results of stability study of optimized batch (F22).

Evaluation parameters	Initially	After 30 days
Weight variation (*n* = 10)	430 ± 1.2	429 ± 3.3
Diameter (mm)	10.0 ± 0.05	10.0 ± 0.05
Thickness (mm)	5.0 ± 0.2	5.0 ± 0.1
Hardness (kg/cm^2^)	6.8 ± 0.2	6.7 ± 0.5
Friability (%)	0.49	0.52
% drug content	97.2 ± 3.8	97.0 ± 1.3
*Y*1 (% drug release at 1 hr)	28.56%	30.07%
*Y*2 (% drug release at 6 hr)	61.81%	60.37%
*Y*3 (% drug release at 12 hr)	95.72%	93.88%
